# Characterization of the complete chloroplast genome of *Achnatherum pekinense* (Poaceae), a widespread weed

**DOI:** 10.1080/23802359.2022.2054377

**Published:** 2022-03-25

**Authors:** Shao-Qiu Xie, Bei Zhong, Bo-Qiang Tong, Shou-Jin Fan

**Affiliations:** a Key Lab of Plant Stress Research, College of Life Sciences, Shandong Normal University, Ji’nan, China; b Shandong Museum, Ji’nan, China; c Shandong Provincial Center of Forest and Grass Germplasm Resources, Ji’nan, China

**Keywords:** *Achnatherum pekinense*, chloroplast genome, phylogenomics

## Abstract

*Achnatherum pekinense* belongs to Poaceae. The complete chloroplast genome of *A. pekinense* was reported in this study. The chloroplast genome was 137,837 bp in size with a canonical quadripartite structure, including two inverted repeat regions (IR) of 21,635 bp for each, a large single-copy (LSC) region of 81,787 bp in length, and a small single-copy (SSC) region of 12,780 bp in length. The overall guanine-cytosine (GC) content of this chloroplast genome was 38.8%, and the corresponding values of the LSC, SSC, and IR regions were 36.9%, 33.1%, and 44.1%, respectively. A total of 113 unique genes were annotated in this chloroplast genome, including four rRNA genes, 31 tRNA genes, and 78 protein-coding genes. The phylogenetic analysis showed that *A. pekinense* was clustered with *A. inebrians*.

*Achnatherum pekinense* [(Hance) Ohwi 1877] is a perennial herb of Gramineae, which is widely distributed on hillsides, grasslands, forests, beaches, and roadsides in northern China at an altitude of 350–1500 meters. At present, the study of *A. pekinense* mainly focused on leaf epidermal structure and powdery mildew (Chu and Yang 1991; Chen et al. 2018). From the perspective of genome, there are few studies on *Achnatherum* species. To date, *Achnatherum* chloroplast genomes are available for only two representatives, *A. splendens* and *A. inebrians* (Li et al. 2019; Wei et al. 2021). There are still many questions related to its phylogeny and species identification on *Achnatherum*. In this study, the chloroplast genome of *A. pekinense* was reported and its phylogenetic position was determined, which will contribute to the study of *Achnatherum*.

Fresh leaves were collected from Shandong Forest and Grass Germplasm Resources Center (Shandong, China 36°37′33.58″N, 117°9′58.97″E). The voucher specimen was deposited at College of Life Sciences, Shandong Normal University (Shou-Jin Fan, e-mail: fansj@sdnu.edu.cn) under the voucher number SD469. Total genomic DNA was extracted by using the modified CTAB method (Doyle and Doyle 1987), and was sequenced by the Novaseq platform at Novogene (Beijing, China). The chloroplast genome assembly was performed with Getorganelle (Jin et al. 2020). The annotation of the chloroplast genome was performed with Plastid Genome Annotator (PGA) (Qu et al. 2019), and then manually corrected with Geneious v9.1.4 (Kearse et al. 2012). The sequence of complete chloroplast genome has been submitted to GenBank under accession number MZ680617.

The complete chloroplast genome of *A. pekinense* was 137,837 bp in length. The overall guanine-cytosine (GC) content was 38.8%. This chloroplast genome contained a total of 113 unique genes, including 78 protein-coding genes (PCGs), 31 transfer RNA genes (tRNAs), and four ribosomal RNA genes (rRNAs). A total of 10 PCGs contained introns, of which eight PCGs (*atpF*, *ndhA*, *ndhB*, *petB*, *petD*, *rpl16*, *rpl2*, and *rps16*) contained one intron and two PCGs (*rps12* and *ycf3*) contained two introns.

A maximum-likelihood (ML) tree was reconstructed to determine the phylogenetic relationships of *A. pekinense* by using RAxML v8.2.10 (Stamatakis 2014), with tree robustness assessment with 1000 rapid bootstrap replicates, and the substitution model was GTRGAMMA. Alignment of 78 shared PCGs was conducted by using MAFFT v7.313 (Katoh and Standley 2013). ML phylogenetic analysis showed that *A. pekinense was* sister to *A. inebrians* (Figure 1).

**Figure 1.**
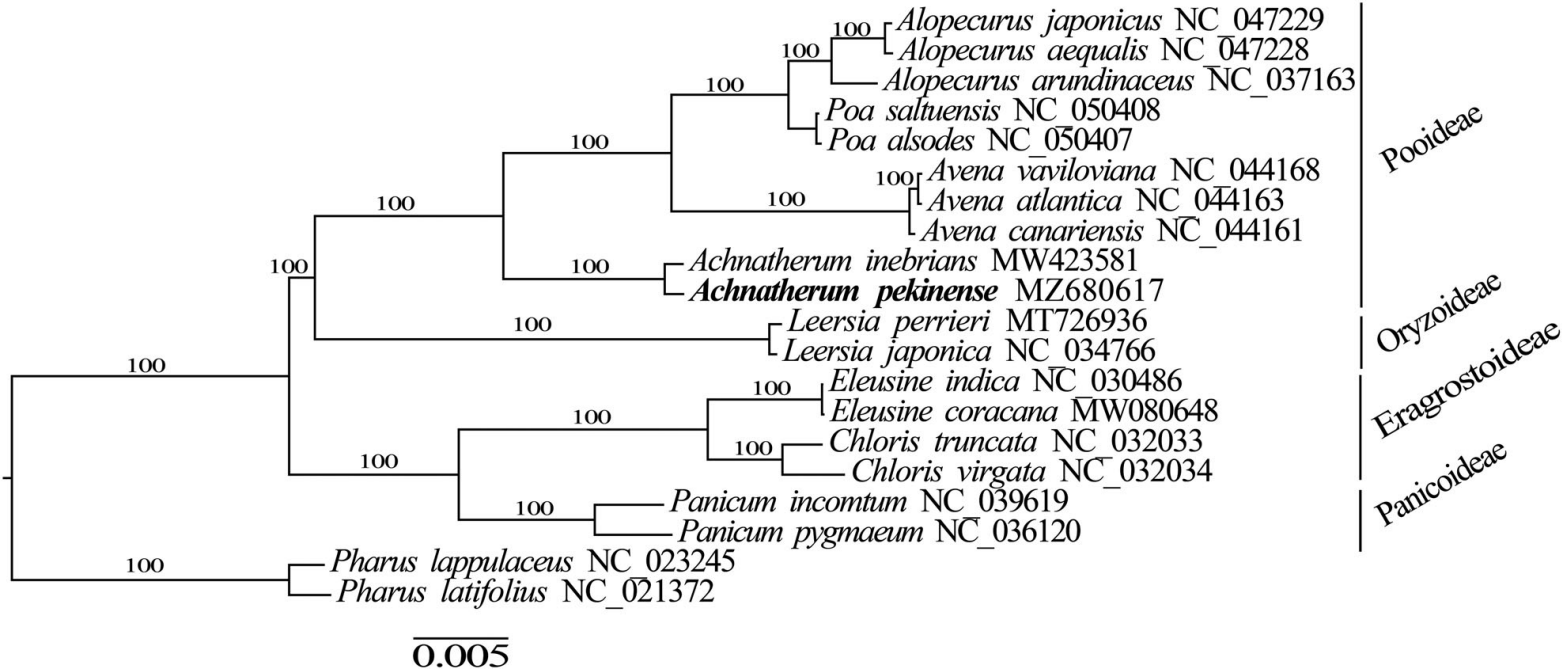
A maximum-likelihood (ML) tree inferred from 78 protein-coding genes is shown. *Pharus lappulaceus* and *Pharus latifolius* are used as outgroup. The numbers on branches are bootstrap support values.

## Data Availability

The genome sequence data that support the findings of this study are openly available in GenBank of NCBI at https://www.ncbi.nlm.nih.gov/ under the accession no. MZ680617. The associated BioProject, SRA, and Bio-Sample numbers are PRJNA751256, SRR15316867, and SAMN20513650, respectively.
